# Medical device-vigilance in Tunisian Center Est University Hospital: knowledge, attitudes and practices of medical staff

**DOI:** 10.1186/2047-2994-4-S1-P269

**Published:** 2015-06-16

**Authors:** M Mohamed, N Bouafia, W Bannour, R Hellali, Z Nawel, A Asma, N Mansor

**Affiliations:** 1 Hospital Hygiene, university hospital Farhat Hached Sousse Tunisia, Jawhara, Tunisia

## Introduction

In the framework of better risks management in hospital environment and in perspective to improve quality and safety care, university hospital center Farhat Hached Sousse (Tunisia) has developed a medical device-vigilance system in order to monitor incidents or risks of incidents that may arise through using medical devices allowed-on in market.

## Objectives

Our objective is to determine medical staff knowledge’s, attitudes and practices in university hospital center Farhat Hached Sousse (Tunisia) regarding medical device-vigilance system establishment.

## Methods

We conducted a descriptive study, type KAP (knowledge’s, attitudes, and practices), in December 2014, among all medical staff exercising at university hospital center Farhat Hached Sousse (Tunisia) who are in direct contact with medical devices. Measuring instrument used is a self-administered questionnaire, preestablished, and pretested. Seizure and data analysis was made by the SPSS software 20.0.

## Results

More than half of participating physicians do not know, nor institution correspondent local (69,5 %(IC 95 % (60 to 77,9 %))), neither existence of standardized form for reporting (56,8% (IC 95 % (47,4 to 66,3%))). Concerning attitudes, majority of investigations (89.5 % (IC 95 % (83,2 at 94,7 %))) shall notify interest of creating medical device-vigilance system. Participants in study report their desire to receive more information about medical device-vigilance system but they relate their desires to follow a training (57.9 % (IC 95% (47,4 à 67,4))).

## Conclusion

Our study highlights lack of information and training in field yet sensitive and heavily regulated. This needs to affirm medical nature relatively to medical device-vigilance by integrating it into health care professional’s curriculum study but also by strengthening awareness and communication around medical device-vigilance system. Success system’s functionality must be supported by promulgation laws and regulations and better organization of regulatory agencies.

## Disclosure of interest

None declared.

